# CYSTIC PANCREATIC LESIONS: IMAGING VERSUS ANATOMOPATHOLOGICAL FINDINGS-HOW TO IMPROVE DIAGNOSTIC ACCURACY?

**DOI:** 10.1590/0102-672020230017e1735

**Published:** 2023-05-26

**Authors:** Rafael Mello Fontolan Vieira, Arthur Soares de Souza, Leandra Ernst Kerche

**Affiliations:** 1 Cancer Regional Hospital, Hepatobiliopancreatic Oncological Digestive Surgery – Presidente Prudente (SP), Brazil;; 2 Faculty of Medicine, Radiology Unit – São José do Rio Preto (SP), Brazil; 3 Faculty of Medicine of Oeste Paulista, Functional Sciences Department – Presidente Prudente (SP), Brazil.

**Keywords:** Pancreatic cyst, Tomography, x-ray computed, Magnetic resonance imaging, Pancreatic neoplasms, Cisto pancreático, Tomografia computadorizada por raios x, Imageamento por ressonância magnética, Neoplasias pancreáticas

## Abstract

**BACKGROUND:**

Pancreatic cystic lesions are a group of pancreatic neoplasms with different behavior and risk of malignancy. Imaging diagnosis and differentiation of these lesions remain a challenge.

**AIMS:**

The aim of this study was to evaluate the agreement between computed tomography and/or magnetic resonance imaging and post-operative pathologic diagnoses of Pancreatic cystic lesions in a University Hospital of São Paulo State.

**METHODS:**

A total of 39 patients with surgically diagnosed Pancreatic cystic lesions were enrolled, as a study cohort from 2009 to 2019. Preoperative radiological and final pathological diagnosis was correlated to measure computed tomography and/or magnetic resonance imaging diagnostic. Pancreatic adenocarcinoma, choledochal pancreatic cyst, mucinous cystadenoma, serous cystadenoma, intraductal papillary mucinous neoplasms, and pancreatic pseudocyst were classified as neoplastic cysts.

**RESULTS:**

It was noted that 27 patients (69.23%) had preoperative computed tomography and magnetic resonance imaging, 11 patients (28.20%) had preoperative computed tomography only, and 1 patient had preoperative magnetic resonance imaging only. The values for diagnoses made only with computed tomography (p=0.47) and from the combination of computed tomography+magnetic resonance imaging (p=0.50) did also point to moderate agreement with the anatomopathological findings. The values pointed to a fair agreement for the diagnosis of mucinous cystadenoma (p=0.3), moderate agreement for intraductal papillary mucinous neoplasms (p= 0.41), good agreement for serous cystadenoma (p=0.79), and excellent agreement for choledochal pancreatic cyst (p=1), pancreatic pseudocyst (p=0.84), and Frantz tumor (p=1) (p<0.05).

**CONCLUSIONS:**

The findings of computed tomography and/or magnetic resonance imaging have an equivalent diagnostic agreement with an anatomopathological diagnosis for differentiating benign from malignant Pancreatic cystic lesions and in suggesting a specific diagnosis. There is no statistical difference between the use of computed tomography alone and computed tomography+magnetic resonance imaging in the improvement of diagnostic accuracy.

## RESUMO

**RACIONAL:**

Lesões císticas pancreáticas são um grupo de neoplasias pancreáticas com diferentes comportamentos e riscos de malignidade. O diagnóstico por imagem e a diferenciação dessas lesões constituem um desafio.

**OBJETIVOS:**

Avaliar a concordância entre o diagnóstico de imagem pré operatório obtido através da tomografia computadorizada e/ou da ressonância nuclear magnética e o diagnóstico anátomopatológico das lesões císticas pancreáticas, no Hospital de Base de São José do Rio Preto – SP.

**MÉTODOS:**

Trinta e nove pacientes com lesões císticas pancreáticas, comprovados cirurgicamente, foram incluídos para o estudo, de 2009 a 2019. O diagnóstico radiológico pré-operatório e o diagnóstico anatomopatológico final foram correlacionadas para medir a acurácia da tomografia computadorizada e/ou da ressonância nuclear magnética. O adenocarcinoma pancreático, o cisto pancreato-coledociano, o cistoadenoma mucinoso, o cistoadenoma seroso, a neoplasia mucinosa papilar intraductal, e o pseudocisto pancreático foram classificados como cistos neoplásicos. As informações foram comparadas e estatisticamente analisadas.

**RESULTADOS:**

Vinte e sete pacientes fizeram tomografia computadorizada e ressonância nuclear magnética pré-operatórios (69,23%), 11 pacientes fizeram apenas tomografia computadorizada (28,20%), e 1 paciente fez apenas ressonância nuclear magnética (2,57%). Os achados de tomografia computadorizada para diagnóstico (p=0,47) e para a combinação tomografia computadorizada+RM (p=0,50) mostraram moderada concordância com os achados anatomopatológicos. Houve leve concordância para o diagnóstico de cistoadenoma mucinoso (p=0,3), moderada concordância para intraductal papillary mucinous neoplasms (p=0,41), boa concordância para cistoadenoma seroso (p=0,79) e excelente concordância para pseudocisto pancreático (p=0,84), cisto pancreato-coledociano (p=1) e tumor de Frantz (p=1) (p<0,05).

**CONCLUSÕES:**

Os achados de imagem da tomografia computadorizada e/ou ressonância nuclear magnética apresentaram concordância diagnóstica com os exames anatomopatológicos na diferenciação de lesões císticas pancreáticas benignas e malignas, porém a diferença entre o uso apenas da tomografia computadorizada e tomografia computadorizada+ressonância nuclear magnética na melhora da acurácia diagnóstica não apresentou relevância estatística tal como a literatura.

## INTRODUCTION

Pancreatic cystic lesions (PCLs) are a heterogeneous group of pancreatic neoplasms that include intraductal papillary mucinous neoplasms (IPMN), the most common, mucinous cystic neoplasms (MCN), including mucinous cystadenoma (MCA), serous cystic neoplasms (SCN), including serous cystadenoma (SCA), and other rare cystic lesions, such as cystic neuroendocrine tumors (cNET), and solid pseudopapillary neoplasms (SPN) that include Frantz tumor, all of which present their own clinical, radiological, and pathological features.^5,6,8,34,35^ Most of these lesions are incidentally discovered due to, most importantly, the widespread and frequent use of abdominal cross-sectional imaging.^10,32^ However, the identification of these lesions remains a problem given the lack of stringent mechanisms to differentiate malignant, benign, and inflammatory lesions^24^.

In some PCLs, such as MCN, radiological assessment plays a major role in the management and risk stratification. Radiology should be able to estimate the level of malignancy in these tumors based on management algorithms that use the presence of high-risk stigmata and worrisome features to propose timelines of follow-up and recommendations of treatment ^12,35^.

CT, magnetic resonance imaging (MRI) and magnetic resonance cholangiopancreatography, positron emission tomography (PET), and PET superseded by fused imaging with CT (PET/CT) are the radiological modalities more frequently used to image pancreatic cysts^1,24^. Once pancreatic lesions are detected on CT or MRI, endoscopic ultrasound (EUS) can also be used for further characterization, as it is a valuable tool for showing internal structures such as septa and mural nodules^15^. However, although cross-sectional imaging modalities constitute a mainstay in the characterization of PCLs, one-third of the cases are incorrectly diagnosed even in high-volume centers and regardless of the use of EUS^3^.

Therefore, the aim of this study was to evaluate the agreement between CT and/or MRI imaging and post-operative pathologic diagnosis of PCL for the first time in a hospital in São Paulo State. In this study, EUS was not included since this imaging modality was introduced in this hospital in the year of 2019.

## METHODS

### Patients

Patients who underwent surgical resection for PCLs at Base Hospital of São José do Rio Preto (São Paulo) were enrolled as study patients from 2009 to 2019. PCLs under the clinical impression of main duct or mixed type of IPMNs were excluded because they are relatively easy to be distinguished from other types of cystic lesions and they could be resected undoubtedly as premalignant lesions. Therefore, 39 patients with surgically proven PCLs were enrolled as a final study cohort in the Base Hospital of São José do Rio Preto. The medical records including age, sex, radiographic, surgical, and pathological data were reviewed. The study protocol was approved by the Ethics Committee of the Hospital, and informed consent was waived under No. 12489519.2.0000.5414.

### Preoperative and postoperative diagnoses

Twenty-seven patients (27/39, 69.23%) had preoperative CT and MRI, 11 patients (11/39, 28.20%) had preoperative CT only, and 1 patient had preoperative MRI only. Imaging studies were read by experienced radiologists correlated to the patients’ clinical data. Pancreatic adenocarcinoma (PAC), choledochal pancreatic cyst (CPC), MCA, SCA, IPNM, pancreatic pseudocyst (PPC), and Frantz tumor were classified as neoplastic cysts. The postoperative diagnosis was made by experienced pathologists using histological and immunohistochemistry methods. The CT examinations were conducted using a Philips Select Brilliance 16 slice (Philips Healthcare, Andover, MA, USA), and MRI examinations were conducted using a Philips Intera 1.5 T and Philips Ingenia 1.5 T (Philips Healthcare, Andover, MA, USA).

### Statistical analysis

The results of preoperative CT and/or MRI and final pathology were compared. The coefficients (95% confidence intervals) were calculated for comparison between imaging diagnoses versus anatomopathological diagnoses, and the values greater than 0 indicate positive agreement, being 0–0.2, poor agreement; 0.21–0.40, fair agreement; 0.41–0.60, moderate agreement; 0.61–0.80, good agreement; and greater than 0.81, excellent agreement^18^. The chi-square test was used to assess the association between agreement and the type of imaging method used. All analyses were performed with the statistical software Minitab^®^ (State College, Pennsylvania, USA), and p<0.05 indicates statistical significance.

### RESULTS

#### Baseline characteristics and final diagnosis of the patients

The baseline characteristics of 39 patients are described in Table 1 (mean age 54±12 years and 61.5% were female). According to the final diagnoses, the most common cysts were PPC (43.9% in anatomopathological diagnoses, 17/39; 41% in imaging diagnoses, 16/39), and among pathologically confirmed malignant cysts, malignant IPNM were most common (15.4% in anatomopathological and imaging diagnosis, 6/39). CT in combination with MRI was used as imaging method in 69.23% of cases (27/39), and caudal body pancreatectomy (30.8%, 12/39) and pseudocyst shunt (25.6%, 10/39) surgeries covered more than half of all surgeries performed.

**Table 1:** Baseline characteristics of patients according to final diagnoses (n=39).

Age, years		54 (44–62)
Sex, female (%)		24 (61.5)
Image examination (%)		
	MRI (%)	1 (2.6)
	CT (%)	11 (28.2)
	CT + MRI (%)	27 (69.2)
Diagnostic Imaging (%)		
	Undetermined complex cyst	4 (10.3)
	Nonspecific pancreatic cyst	1 (2.6)
	CPC	1 (2.6)
	MCA	7 (17.9)
	SCA	3 (7.7)
	IPNM	6 (15.4)
	PPC	16 (41)
	Frantz tumor	1 (2.6)
Anatomopathological diagnosis (%)		
	PAC	5 (12.8)
	CPC	1 (2.6)
	MCA	7 (17.9)
	SCA	2 (5.1)
	IPNM	6 (15.4)
	PPC	17 (43.6)
	Frantz tumor	1 (2.6)
Surgery (%)		
	CT-guided biopsy	6 (15.4)
	Bilio-digestive shunt	1 (2.6)
	Pseudocyst shunt	10 (25.6)
	Endoscopic drainage	2 (5.1)
	Exploratory laparotomy	3 (7.7)
	Caudal pancreatectomy	12 (30.8)
	Total pancreatectomy	1 (2.6%)
	Whipple	4 (10.2%)

CT: computed tomography; MRI: magnetic resonance imaging; CPC: choledochal pancreatic cyst; PAC: pancreatic adenocarcinoma; MCA: mucinous cystadenoma; SCA: serous cystadenoma; IPMN: intraductal papillary mucinous neoplasms; PPC: pancreatic pseudocyst.

#### Comparison of agreement between imaging and pathologic diagnoses

Table 2 describes the concordance between imaging and anatomopathological findings. The imaging diagnoses for indeterminate complex cyst and nonspecific pancreatic cyst did not show any agreement, as well as the anatomopathological diagnosis for PAC, observed for five patients, and which was not previously identified in CT and/or MRI. On the other hand, all other possible diagnoses had an agreement between imaging and anatomopathological diagnoses of at least 50%, with exception of MCA (42.9% of agreement), and PPC being predicted by imaging in 93.8% of the cases.

**Table 2:** Agreement between image diagnosis and anatomopathological diagnosis.

Imaging diagnosis	Anatomopathological diagnosis						
	Pancreatic adenocarcinoma (%)	Coledochal pancreatic cyst (%)	Mucinous cystadenoma (%)	Serous cystadenoma (%)	IPMN (%)	Pancreatic pseudocyst (%)	Frantz tumor (%)
Undetermined complex cyst	75 (3)	0 (0)	0 (0)	0 (0)	0 (0)	25 (1)	0 (0)
Nonspecific pancreatic cyst	0 (0)	0 (0)	0 (0)	0 (0)	100 (1)	0 (0)	0 (0)
Pancreatic-choledocean cyst	0 (0)	**100 (1)**	0 (0)	0 (0)	0 (0)	0 (0)	0 (0)
Mucinous cystadenoma	14.3 (1)	0 (0)	**42.9 (3)**	0 (0)	28.6 (2)	14.3 (1)	0 (0)
Serous cystadenoma	0 (0)	0 (0)	33.3 (1)	**66.7 (2)**	0 (0)	0 (0)	0 (0)
IPMN	16.7 (1)	0 (0)	33.3 (2)	0 (0)	**50 (3)**	0 (0)	0 (0)
Pancreatic pseudocyst	0 (0)	0 (0)	6.3 (1)	0 (0)	0 (0)	**93.8 (15)**	0 (0)
Frantz tumor	0 (0)	0 (0)	0 (0)	0 (0)	0 (0)	0 (0)	**100 (1)**

IPMN: intraductal papillary mucinous neoplasm.

Values in bold were consistent. Percentages are calculated in relation to the totals of the lines.

The agreement for diagnosis using only CT was 72.73% and for those using CT+MRI was 59.26%. The overall agreement was 64.10%. Besides the differences in agreement based on the imaging methods used, there were no significant differences between them according to the chi-square test (p=0.551) (Table 3).

**Table 3:** Percentages of agreement and kappa-concordance coefficients for imaging and anatomopathological diagnoses, according to the type of image examination performed

Comparison	Agreement % (95%CI)	Kappa (95%CI)	Kappa p-value
Global	64.10 (47.18–78.80)	0.53 (0.38–0.67)	<0.001*
Tomography	72.73 (39.03–93.98)	0.47 (0.13–0.8)	0.0032*
Tomography+MRI	59.26 (38.80–77.61)	0.50 (0.33–0.66)	<0.001*

CI: confidence interval; MRI: magnetic resonance imaging.

* Statistically significant p-values at a level of 5%.

Table 3 shows the results of the Cohen's kappa test, presenting a p-value=0.528 (p<0.001) with a moderate agreement between the imaging and anatomopathological diagnoses. The values for diagnoses made only with CT (p=0.47) and from the combination of CT+MRI (p=0.50) did also point to a moderate agreement with the anatomopathological findings.

The only diagnosis (of SCA) made from only MRI was not included in the analysis given in this table; however, it was in agreement with the anatomopathological finding. P-value for the chi-square test, referring to the association between the type of examination and the occurrence of agreement, is equal to 0.5513 (p>0.05).

The agreement between imaging and anatomopathological diagnoses based on the type of diagnosis was analyzed. The p-values pointed to a fair agreement for the diagnosis of MCA (p=0.3), moderate agreement for IPMN (p=0.41), good agreement for SCA (p=0.79), and excellent agreement for CPC (p=1), PPC (p=0.84), and Frantz Tumor (p=1) (p<0.05).

### DISCUSSION

A distinction between the different types of PCLs is very important as the malignant potential of PCL varies between their various types. While IPMN, MCN, SPN, and cNET are premalignant cysts and require surveillance or surgical resection, SCN are mostly benign^8,25,35^. However, the longitudinal risk of malignancy of the latter type of cysts is very limited as there is a lack of studies and reports on the natural history of these PCLs^5^.

The risk of advanced malignant neoplasia in IPMN is highly elevated when the main duct is involved (36–100%) as it increases the risk of pancreatic ductal adenocarcinoma (PDAC)^4,9,36,37^. The risk of advanced neoplasia in MCN has been shown to be 10–39%^11,13,27,31,40^. It has been reported invasive cancer in 15% of the resected SPN^19^ and 10% of cNET^17^.

CT and MRI are the mainstay of assessment of PCL^30^, and a European experts’ consensus recommended that CT and/or MRI should be performed in all patients with PCLs^5^. However, previous studies have shown that preoperative diagnosis of PCL by CT/MRI was incorrect in one-third of the cases, even in experienced high-volume centers^3,6^. In this study, 39 patients who underwent surgical resection for PCL were analyzed to compare the agreement between the imaging and anatomopathological diagnoses.

The overall agreement between the diagnoses was 64.10%. CT alone had a higher agreement to anatomopathological diagnosis (72.73%) when compared to CT+MRI (59.26%) contradicting other studies that showed that CT+MRI had higher accuracy in PCL diagnosis^13,19^. However, the chi-square test showed that there was no statistical difference between CT+MRI and CT alone and both methods showed moderate agreement (p=0.528) between the imaging and anatomopathological findings.

The different types of PCLs have morphological differences that can be helpful in the imaging diagnosis. For IPMN, according to the Fukuoka Guidelines, the location and the involvement of the main duct can be used for morphological classification as main duct (MD), side branch (SB), and mixed type (MT). Usually, MD-IPMN causes an abrupt dilation of the main pancreatic duct and SB-IPMN causes dilation of side branches of the main pancreatic duct. MT-IPMN meets both criteria for MD-IPMN and SB-IPMN^33^. IPMNs exhibit a spectrum of neoplastic transformation that ranges from adenomas, actually named low-grade dysplasia, to invasive carcinomas, actually named high-grade dysplasia as outlined by World Health Organization (WHO)^2,9^.

MCN are mainly unilocular or septated macrocystic cysts and normally arise in the body and tail of the pancreas^14,28,40^. SCN can be divided into macro and microcystic, mixed macro and microcystic, and solid SCN^7,16^. Macrocystic SCN are composed of few but large cysts, and it can be very difficult to distinguish from MCN or SB-IPMN. Microcystic SCN are composed of multiple small cystic spaces, and a central calcification or scar can be present^21^. Solid SCN can be difficult to differentiate from SPN that usually appear as a mixed cystic and solid mass in the pancreas^26^. cNET can be mostly visualized as a mixed cystic and solid mass in the pancreas, and a heterogeneous enhancement can appear due to necrotic and hemorrhagic changes^22,23^.

The agreement between the imaging and anatomopathological diagnoses based on the different types of cysts was a fair agreement for the diagnosis of MCA (p=0.3), moderate agreement for IPMN (p=0.41), good agreement for SCA (p=0.79), and excellent agreement for CPC (p=1), PPC (p=0.84), and Frantz tumor (p=1) (p<0.05) in concordance with other studies^20,39^. These studies also corroborate the fact that there is no statistically significant difference between CT and MRI.

The agreement found in this study for the different types of PCLs shows that imaging diagnosis can be a very important tool to identify and follow up premalignant cysts. It can also be used to identify pancreatic cysts that take several years to become invasive cancers, such as IPMN and MCN, offering opportunities for early detection and surgical cure. It can also be used to avoid unnecessary surgeries as in the case of SCN that are completely benign.

This study has some important limitations as it is a retrospective study conducted at a single center with a low number of patients. This low number of patients occurred as only patients who underwent CT and/or MRI and had pathological confirmation of their PCL type were included. However, this is a very important study, as it composes a very few number of studies that has compared the diagnostic value of CT and/or MRI in the evaluation of various cystic lesions of the pancreas. Also, the lack of statistical difference between CT and MRI could occur because the imaging devices used in this hospital may not be the top-of-the-line, in addition to the greater experience of the professionals to analyze the CT results instead of MRI.

## CONCLUSIONS

This study shows that CT and/or MRI have a statistically equivalent diagnostic agreement with an anatomopathological diagnosis for differentiating benign from malignant PCL and in suggesting a specific diagnosis. There is no statistical difference between the use of CT alone and CT+MRI in the improvement of diagnostic accuracy.
